# First record of the subgenus *Damaeus* (*Paradamaeus*) Bulanova-Zachvatkina (Oribatida, Damaeidae) from China, with description of a new species

**DOI:** 10.3897/zookeys.160.2160

**Published:** 2011-12-29

**Authors:** Lixia Xie, Yi Yan, Rong Huang, Maofa Yang

**Affiliations:** 1 Institute of Entomology, Guizhou University; The Provincial Key Laboratory for Agricultural Pest Management of Mountainous Region, Guiyang, Guizhou, P. R. China, 550025

**Keywords:** Oribatida, *Damaeus*, *Paradamaeus*, new species, new record

## Abstract

A new species *Damaeus (Paradamaeus) yushuensis* **sp. n.** is described from Three Rivers’ Headwaters National Natural Reserve, Qinghai province, China. This is the first record of this subgenus in China. A key is given to distinguish all species of the genus.

## Introduction

Bulanova-Zachvatkina (1957) proposed *Paradamaeus* to be a subgenus of *Damaeus* with *Damaeus (Paradamaeus) clavipes* Hermann, 1804 as type species, which is only single known species in the world. The diagnosis of this subgenus as given by Bulanova-Zachvatkina (1957): large body size; rugged prodorsum with distinct ridges; apophysis *P* well developed; interlamellar setae(*in*) much shorter than sensillus (*ss*); prodorsal tubercles *Da*, *Ba* and *Bp* present; spinae adnatae (*s*.*a*.) well developed; companion seta *d* lost on genu III; setation of genua 4-4-2-3.

We firstly recorded the species of *Paradamaeus* from Three Rivers’ Headwaters National Natural Reserve, Qinghai, China, which represented as new species, *Damaeus (Paradamaeus) yushuensis*. A key to the identification of all known species of *Damaeus* (*Paradamaeus*) is given.

## Material and methods

Measurements and descriptions are based on specimens mounted in temporary cavity slides that were studied using a standard light microscope equipped with a drawing tube. In figures we used the following abbreviations: prodorsal and sejugal apophyses (*Ba*, *Bp*), lateral apophyses (*Sa*, *Sp*) and coxisternal apophyses (*E2a*, *E2p*, *Va*, *Vp*), spina adnatum (*s*.*a*.), discidium (*di*), prodorsal setae (*ro*, *le*, *in*, *ex*), bothridium (*bo*), sensillus (*ss*), notogastral or gastronotal setae (*c*-, *l*-, *h*-, *p*- series), adanal and anal setae (*ad*-, *an*- series), aggenital setae (*ag*), coxisternal setae (1*a*, 1*b*, 1*c*, 2*a*, 3*a*, 3*b*, 3*c*, 4*a*, 4*b*, 4*c*, *4d*), opisthonotal gland opening (*gla*), lyrifissures or cupules (*ia*, *im*, *ip*, *ian*, *iad*, *ips*, *ih*).

Terminology generally follows Grandjean (1949, 1954b, 1960), Miko (2006) and Norton and Behan-Pelletier (2009). The unit of measurement is micrometre (μm). All type specimens and other material studied are kept in alcohol and deposited in the Institute of Entomology, Guizhou University, Guiyang, China (GUGC).

## Taxonomy

### Damaeus (Paradamaeus) clavipes

(Hermann, 1804)

Notaspis clavipes : Hermann 1804; Grandjean 1936, 1954a; Hammen 1952; Sellnick 1960.
Oribata clavipes : Oudemans 1900; Kulczyński 1902; Sellnick 1928.
Belba clavipes : Willmann 1931; Grandjean 1935; Schweizer 1956.
Damaeus (Paradamaeus) clavipes : Bulanova-Zachvatkina 1957; Balogh and Balogh 1992; Pérez-Íñigo 1997; Subías 2004; Miko 2006.
Paradamaeus clavipes : Bulanova-Zachvatkina 1967, 1975; Schatz 1983; Siepel 1996.

#### Distribution.

China, Germany, Ireland, Southern Mediterranean, Faroe Islands, Norway, Latvia, Caucasus, Crimea, Ukraina, Czechoslovakia, Czech Republic, Belgium, Sweden, England, America, Finland, Azores Islands, France, Austria, Netherlands.

#### Keys to species of *Damaeus* (*Paradamaeus*)

**Table:** 

1	With dorsosejugal tubercles *Da*, *Dp*; setae *in* short and thin; notogastral setae similar to one another	*Damaeus (Paradamaeus) clavipe*s
–	Without dorsosejugal tubercles *Da*, *Dp*; setae *in* long and thick; notogastral setae not similar to one another	*Damaeus (Paradamaeus) yushuensis* sp. n. (Figs 1–6)

### Damaeus (Paradamaeus) yushuensis sp. n.

urn:lsid:zoobank.org:act:9CA2E39F-5E98-43B2-87D5-DDD3F4119690

http://species-id.net/wiki/Damaeus_yushuensis

**Figure 1.**
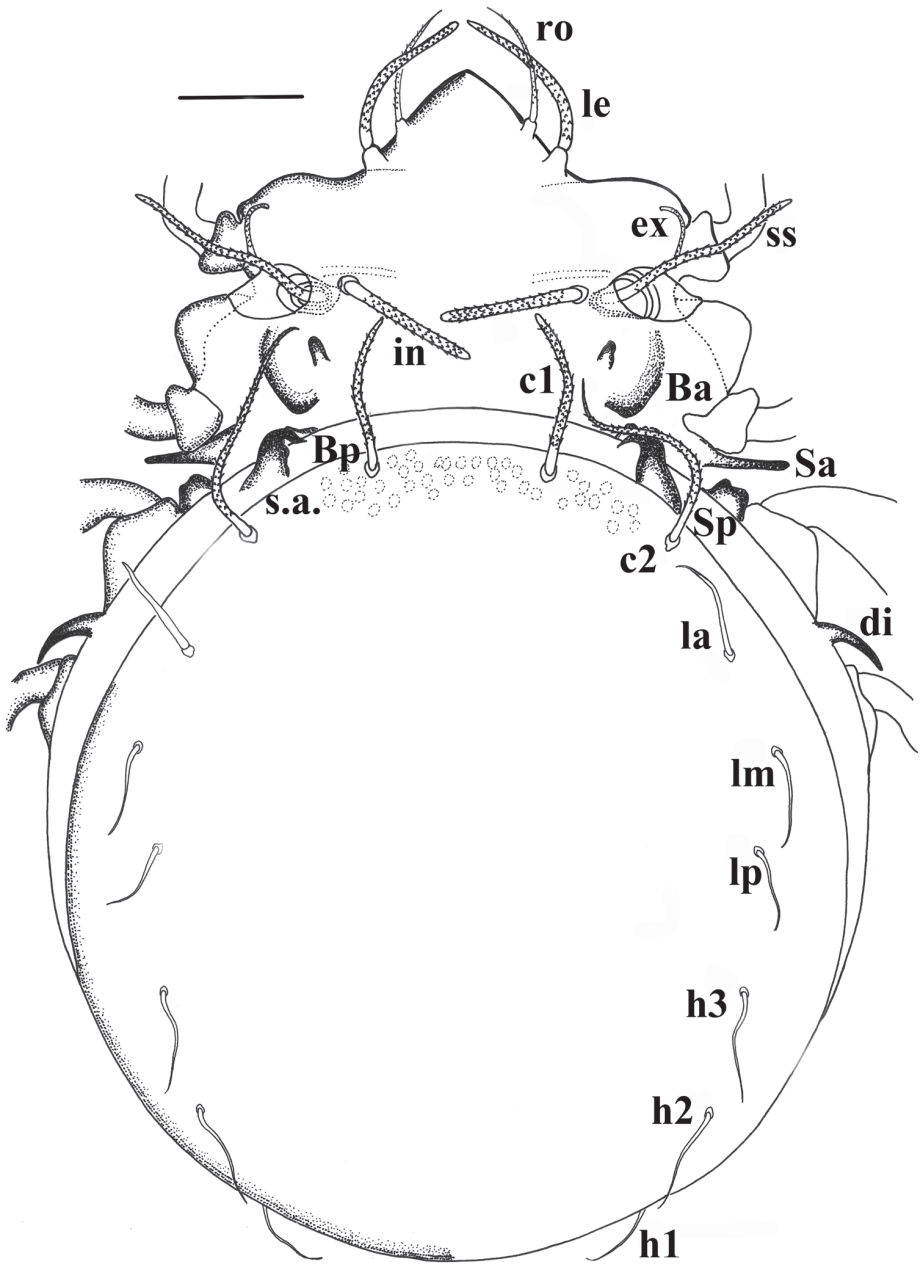
*Damaeus (Paradamaeus) yushuensis* sp. n.– dorsal view (100μm)

Figs 1 [Fig F2] [Fig F3] [Fig F4] [Fig F5] –6 

#### Material examined.

Holotype: male (in alcohol, QHYS-XLX-8-5), China, Three Rivers' Headwaters Natural Reserve Area of Yushu Tibetan Autonomous Prefecture, Qinghai province (32°33'48.65"N , 97°39'55.66"E), from soil under the *Picea crassifolia*, 3464M, 5 Aug., 2009, col. Lixia Xie. Paratypes: Three females (in alcohol, QHYS-XLX-8-5), same data as holotype; Two males (in alcohol, QHYS-XLX-8-6), same data as QHYS-XLX-8-5, from soil under the *Kobresia pygmaea*.

#### Diagnosis.

Propodolateral apophysis *P* distinct, with broader base and arched tip; setae *ro* slightly barbed, setae *le* heavily barbed, thick. Sensillus short, thick, heavily barbed and rod-like. Interlamellar setae long, rather thick and conspicuously barbed. Prodorsal tubercles *Da*, *Dp* absent, *Ba* well developed, *Bp* weakly developed. Spinae adnatae beak like, short, distinct, strongly curved inwards (about 30 µm in total), with broader base and quite sharp tip. Notogastral setae smooth, slender except *c*- series and *p*- series. *Setae of c*- series rather thick, with conspicuously barbed and frizzled tip, oriented forwards and the rest backwards.

Comparative length of notogastral setae: *lp*< *lm*= *ps*3< *ps*2< *la*= *h*_3_= *h*_2_= *h*_1_< *ps*1< *c*_1_<*c*_2_. Epimeral setae mostly smooth except 1*b*, 1*c*, 3*b*, 3c, 4*d* and hypostomal setae *a*, *m*, *h*. Seta 1*a*, 2*a* and 3*a* rather short. Epimeral setal formula: 3-1-3-4. Enantiophyses *E*2 and *V* present, *E*2p and *Vp* weakly developed, *E*2a and *Va* well developed. Parastigmatic tubercle *Sa* long, acuminate, with sharp tip; *Sp* small, triangular. Hypostomal setae *a*, *h* and *m* thin, slightly barbed. Legs rod-like and longer than body.

**Figure 2. F2:**
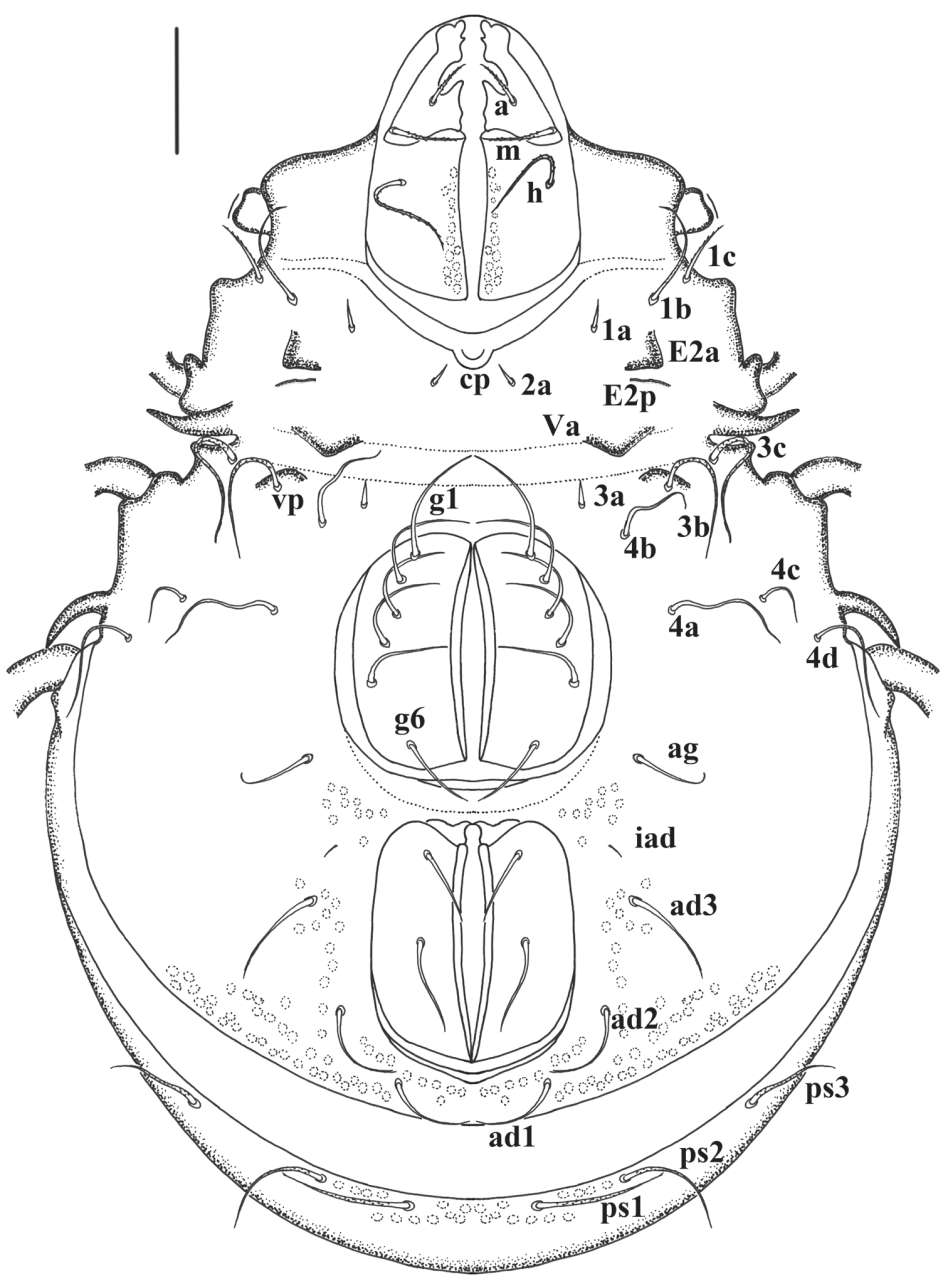
*Damaeus (Paradamaeus) yushuensis* sp. n. – Ventral view (100μm)

**Figure 3. F3:**
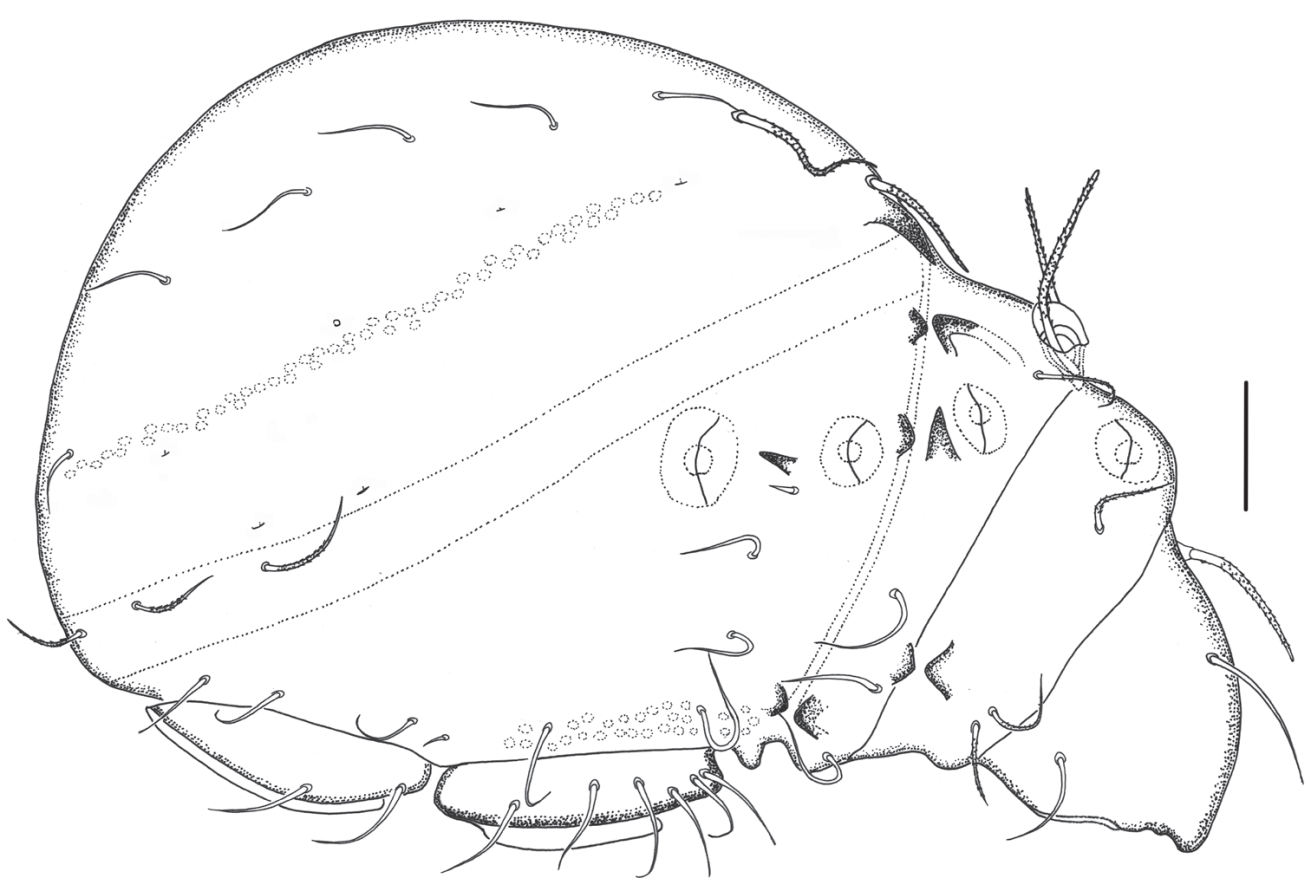
*Damaeus (Paradamaeus) yushuensis* sp. n. – lateral view (100μm)

#### Description of adult.

Dimensions. Holotype: Body length 980; length of prodorsum: 420, width 280, height 370, setae: *ss* 175, *in* 100, *le* 150, *ro* 125, *ex* 75, distance between setae: *ro*-*ro* 110, *in*-*in* 125, *le*-*le* 125, *in*-*le* 105, *le*-*ro* 40; length of notogaster: 700, width 670, height 780; setae: *c*1 135, *c*2 175, *la* 110, *lm* 95, *lp* 90, *h*3 110, *h*2 110, *h*1 110, *ps*1 125, *ps*2 105, *ps*3 95; *c*1*-c*2 75, *c*2*-c*2 275, *la*-*la* 375, *lm*-*lm* 435; ventral region: genito-aggenital plate 225×220, ano-adanal plate 190×175. Paratypes: length of prodorsum: 415-425, width 275-285, height 365-375; length of notogaster: 695-705, width 665-670, height 775-780; ventral region: genito-aggenital plate 220×215, ano-adanal plate 185×170.

**Integument.** Surface of body and leg segments with filamentous cerotegument. Conspicuous microtubercles present on prodorsum and around leg acetabula, legs with dense fungal mycelic*.*

**Prodorsum** (Fig. 1). Triangular, propodolateral apophysis *P* distinct, with broader base and arched tip. Lamellar setae (*le*) and rostral setae (*ro*) both in dorsalaterad position, long, arched and tapered. Lamellar setae with obvious barbs, thick, being longer than slender, weakly barbed rostral pair. Bothridia well developed, funnel-like, with broad margin and pair of thick, heavily barbed sensillus. Interlamellar setae (*in*) long, thick and conspicuously barbed (specially in holotype). Exobothridial setae (*ex*) thick, with obvious barbs, frizzled. Comparative length of prodorsal setae: *ex*< *in*< *ro*< *le*< *ss.* Weakly developed transverseridge connected to the base of bothridium and directed to median end of prodorsum. Prodorsal tubercles *Da* absent, *Ba* distinct; *Bp* weakly developed, usually as tuberculate sclerotised ridge, in light microscope sometimes discernible only in lateral view.

**Notogaster** (Fig. 1). Circular viewed perpendicular to circumgastric scissure, length almost equivalent to wider. Spinae adnatae beak like, short, distinct, strongly curved inwards (about 30 µm in length), with broader base and quite sharp tip. Notogastral setae short, smooth, slender except *c*- series and *p*- series. Setae of *c*- series rather thick and long, with conspicuous barbed and frizzled tip, oriented forwards and the rest backwards. Comparative length of notogastral setae: *lp*< *lm*= *ps*3< *ps*2< *la*= *h*3= *h*2= *h*1< *ps*1< *c*1< *c*2. Pseudanal setae comparatively long, with obvious barbs, attenuate.

**Ventral region.** (Fig. 2). Epimere I with medial pit (*cp*). Epimeral setae mostly smooth except 1*b*, 1*c*, 3*b*, 3*c*, 4*d*; setae 1*c*, 3*b*, 3c, 4*d* long, with obvious barbs; Seta 1*a*, 2*a* and 3*a* rather short, lanciform. Epimeral setal formula: 3–1–3–4. Ano-genital setal formula: 6–1–2–3. Enantiophyses *E*2 and *V* present, *E*2*a* triangular with pointed tip; *E*2*p* weakly developed, usually as tuberculate sclerotised ridge. Ventrosejugal tubercle *Va* large, strong, represented by broad ridge; *Vp* represented by low, broadly curved ridge, with setae 3*b* Parastigmatic tubercle *Sa* long, acuminate, with broader base and heavily pointed tip; *Sp* small, triangular; Discidium (*di*) long, acuminate, with broader base and heavily pointed tip, directed posterolaterad.

**Figure 4. F4:**
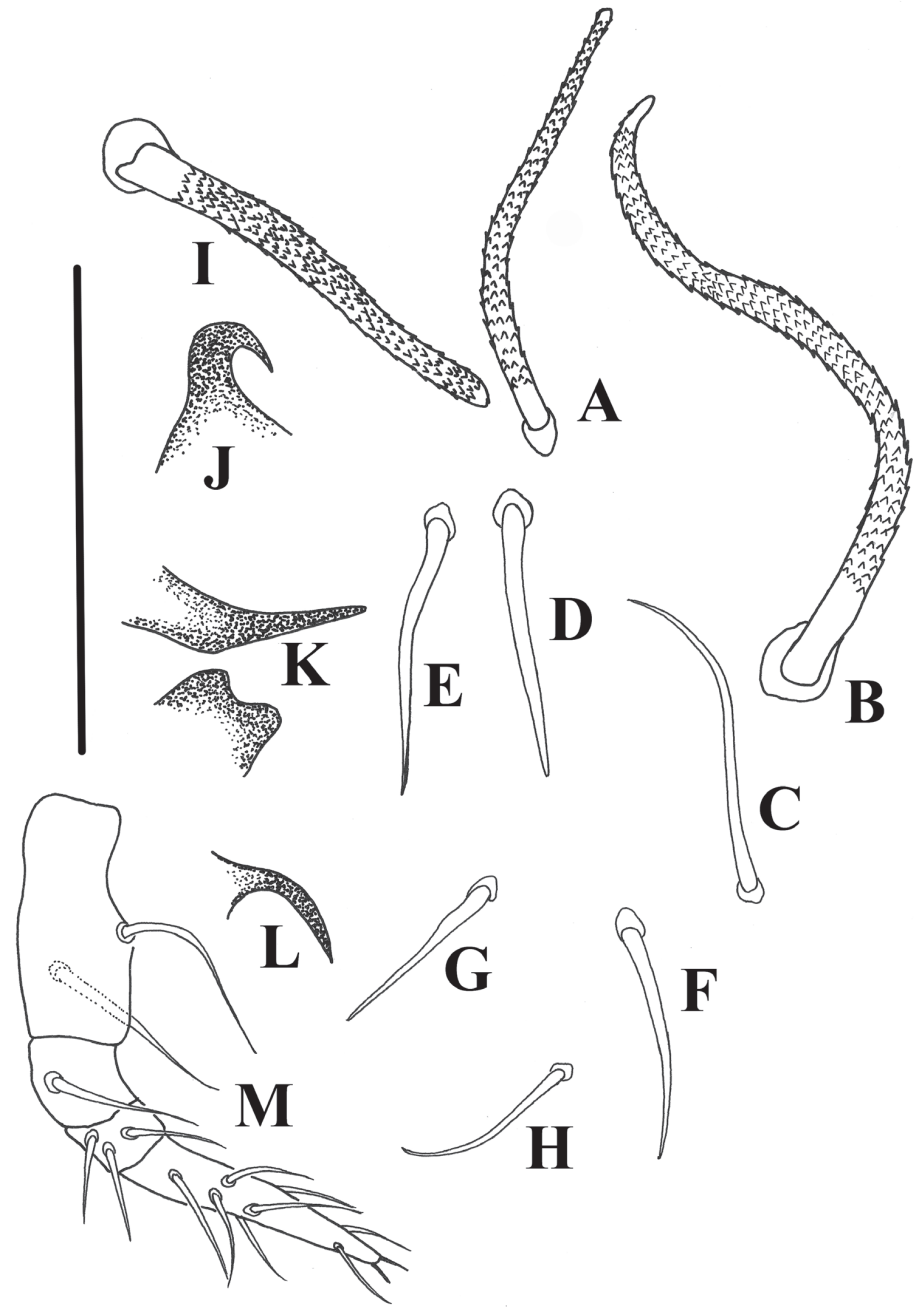
*Damaeus (Paradamaeus) yushuensis* sp. n. **A–H** notogastral setae (100μm) **I** interlamellar seta(100μm) **J** s.a.–spina adnata(100μm) **K** Sa –anterior sejugal apophysis(100μm), Sp –posterior sejugal apophysis(100μm) **L** di –discidium(100μm) **M** Pe. –pedipalp(100μm).

**Gnathosoma.** Infracapitular mentum without noticeable microtubercles. Hypostomal setae *a*, *m*, *h* slender, weakly barbed. Chelicera rather strong, fixed and movable digits with four blunt teeth; setae *cha* with obvious barbsand *chb* smooth. Palpal setation: 0–2–1–3–8, including solenidion *ω* (Fig. 4M).

**Figure 5. F5:**
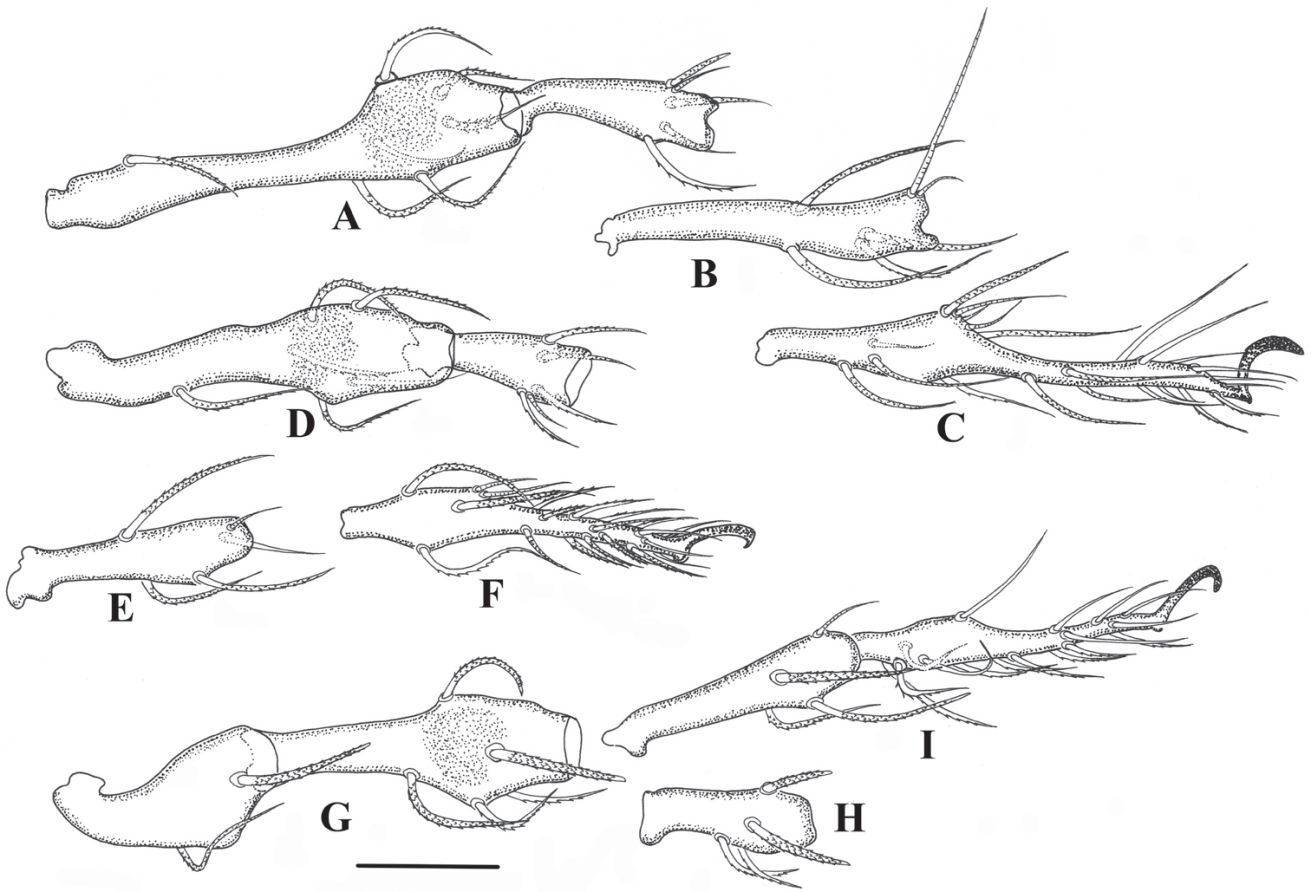
*Damaeus (Paradamaeus) yushuensis* sp. n. **A** femur, genu I (100μm) **B** tibia I (100μm) **C** tarsus I (100μm) **D** femur, genu II (100μm) **E** Tibia II (100μm) **F** tarsus II (100μm) **G** trochanter, femur III (100μm) **H** genu III (100μm) **I** tibia, tarsus III (100μm).

**Figure 6.**
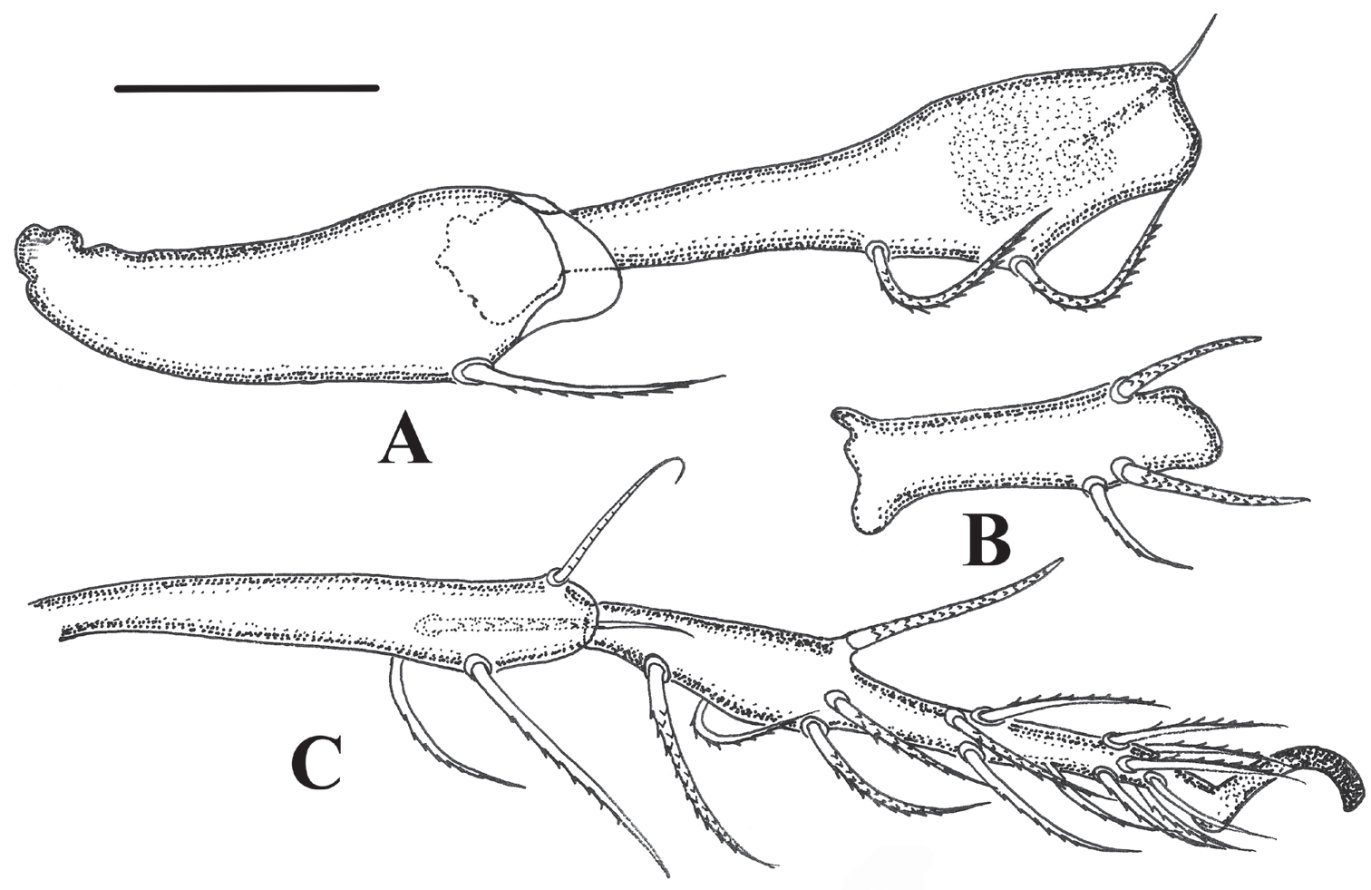
*Damaeus (Paradamaeus) yushuensis* sp. n. **A** trochanter, femur IV (100μm) **B** genu IV (100μm) **C** tibia, tarsus IV (100μm).

**Legs.** (Figs 5–6). Monodactylous, moderately long, leg I, III, IV longer than body, leg II shorter than body. Relative length of femur to tarsus of legs I to IV 1: 0.88: 1.01: 1.2. Leg IV 1.3 times ventral body length. Femur IV 1.4 times length of trochanter IV, proximal stalk 1.4 times length of bulb. Leg setae medium in length and thick, mostly with distinct short barbs on outer curvature. Setal formulas of legs as follows (from trochanter to tarsus, famulus and solenidia included): I: 1–7–4 (1)–4 (2)–20 (2); II: 1–6-4 (1)–4 (1)–18 (2); III: 2–4-3 (1)–3 (1)–16 (0); IV: 1–4-3 (1)–3 (1)–15 (0). Solenidia of genua I-II with companion seta *d*. Solenidia *δ* equivalent to seta *d* on genuaI, Solenidia *δ* shorter and thinner than seta *d* on genuaII. Solenidion *φ*1 on tibia I 3 times longer than *φ*2. Seta *d* absent from all tibiae, solenidia on all tibiae free, as usual for genus.

#### Etymology.

The specific name is derived from the type locality, Yushu Tibetan Autonomous Prefecture, Qinghai province.

#### Remarks.

This new species is characterised by following characters: interlamellar setae (*in*) long, rather thick and conspicuously barbed; spinae adnatae beak like, short, distinct, strongly curved inwards; *c*-series rather thick, long with conspicuous barbed and frizzled tip, oriented forwards and the rest backwards, other notogastral setae smooth, slender and short, except *p*- series (see Table 1).

**Table 1.** Comparison of two species belonging to the subgenus *Paradamaeus*

Morphological character	*Damaeus (Paradamaeus) clavipes*	*Damaeus (Paradamaeus) yushuensis* sp. n.
propodolateral apophysis *P*	with angular tip	with arched tip
Interlamellar setae (*in*)	short, thin	long, thick
Spinae adnatae (s.a.)	slender, medium long, triangular with sharp tip	beak like, short, quite sharp tip
Notogastral setae *c*1, *c*2	similar in length, slender	unequal length, thick
Number of setae on Femora III–IV	5	4
Prodorsal tubercles *Da*, *Dp*	present	absent
Parastigmatic tubercle *Sa*	small, indistinct	large, acuminate, with broader base and heavily pointed tip

## Supplementary Material

XML Treatment for Damaeus (Paradamaeus) clavipes

XML Treatment for Damaeus (Paradamaeus) yushuensis
